# The association between COVID‐19, personal wellbeing, depression, and suicide risk factors in Australian autistic adults

**DOI:** 10.1002/aur.2614

**Published:** 2021-09-21

**Authors:** Darren Hedley, Susan M. Hayward, Kathleen Denney, Mirko Uljarević, Simon Bury, Ensu Sahin, Claire M. Brown, Angela Clapperton, Cheryl Dissanayake, Jo Robinson, Julian Trollor, Mark A. Stokes

**Affiliations:** ^1^ Olga Tennison Autism Research Centre, School of Psychology and Public Health La Trobe University Melbourne Victoria Australia; ^2^ School of Health and Social Development Deakin University Melbourne Victoria Australia; ^3^ School of Social and Political Sciences University of Melbourne Melbourne Victoria Australia; ^4^ School of Psychological Sciences University of Melbourne Melbourne Victoria Australia; ^5^ School of Psychology Deakin University Melbourne Victoria Australia; ^6^ Melbourne School of Population and Global Health University of Melbourne Melbourne Victoria Australia; ^7^ Orygen The National Centre of Excellence in Youth Mental Health Melbourne Victoria Australia; ^8^ Centre for Youth Mental Health University of Melbourne Melbourne Victoria Australia; ^9^ Department of Developmental Disability Neuropsychiatry University of New South Wales Sydney New South Wales Australia

**Keywords:** adults, autism spectrum disorder, COVID‐19 pandemic, depression, gender, suicide, wellbeing

## Abstract

The COVID‐19 pandemic has had a significant impact on the mental health and wellbeing of the world's population, with particularly negative effects on vulnerable populations, including autistic people. Although some consensus regarding specific impact on aspects of wellbeing and mental health in autism is starting to emerge, it is unclear whether the pandemic has increased suicide risk. The goals of this study were to examine (a) potential associations between COVID‐19 impact and depression, personal wellbeing, and suicide risk factors in Australian autistic adults and (b) age and gender effects. The COVID‐19 Impact Scale (CIS), Personal Wellbeing Index, Patient Health Questionnaire, and the Suicide Behavior Questionnaire, Revised (SBQ‐R), were administered to 111 autistic adults aged 20 to 71 years during the second wave of the COVID‐19 pandemic in Australia. COVID‐19 impact showed small associations with poorer personal wellbeing (*r =* −0.224, *p =* 0.023, [−0.409, −0.016]) and higher depressive symptoms (*r =* 0.268, *p =* 0.006, [0.056, 0.445]) and was not associated with the SBQ‐R suicide risk score (*r =* 0.081, *p =* 0.418, [−0.118, 0.264). No significant effects were identified for age. Although model results were similar for women and men, the strength of the associations between personal wellbeing and depression (*z =* −2.16, *p =* 0.015), and depression and SBQ‐R suicide risk (*z =* 1.961, *p =* 0.025), were stronger in women than in men. Qualitative analysis of an open response question from the CIS suggested that the pandemic had both positive and negative impacts on participants. The COVID‐19 pandemic has had a large impact on the mental health and wellbeing of the world's population, particularly vulnerable populations such as autistic people. It is not known if these impacts on mental health and wellbeing have increased suicide risk. Our findings suggest that the impact of the COVID‐19 pandemic may be associated with poorer wellbeing and higher depression, but is not associated with suicide risk. Overall, autistic people reported both positive and negative impacts of the pandemic on their lives.

## 1 INTRODUCTION

The COVID‐19 viral pandemic has dramatically impacted the world's population (Moynihan et al., 2021; Salari et al., 2020; Viner et al., 2020), including significant negative impacts on mental health (Brooks et al., 2020; Cooke et al., 2020; O'Connor et al., 2021; Ramiz et al., 2021). Among the most vulnerable to these impacts are those with pre‐existing mental health conditions (Newby et al., 2020; O'Connor et al., 2021; Tan et al., 2020) and disability (Kavanagh et al., 2021). People diagnosed with Autism spectrum disorder (ASD; henceforth ‘autism’) have a heightened vulnerability to mental ill‐health (Jokiranta‐Olkoniemi et al., 2021; Lai et al., 2019; Uljarević et al., 2020) and therefore may be particularly vulnerable to the impact of COVID‐19 (Baweja et al., 2021; Oomen et al., 2021). Although some benefits have been reported in this population (e.g., reduced stress associated with sensory and social demand, improved sleep), negative impacts such as increased worry (e.g., about access to health services, medication and food, safety and security, work, and pets) and stress (often associated with a loss of routines and social supports) (Corbett et al., 2021; Eshraghi et al., 2020; Lugo‐Marín et al., 2021; Oomen et al., 2021) have been reported. Autistic females, younger adults (<25 years), those with an existing co‐occurring mental health condition, and those with a personal experience with COVID‐19 (e.g., knowing someone with COVID‐19 and testing positive themselves) may be the most significantly impacted (Bal et al., 2021). Given both the impact of COVID‐19 on mental health, and the increased vulnerability of autistic people, it is important to further characterize mental health impacts in this population. Of the most severe possible outcomes associated with mental ill health is concern that the COVID‐19 pandemic may have increased suicide risk (O'Connor et al., 2021). However, this association remains unexplored in the context of autism.

## 2 SUICIDE VULNERABILITY DURING COVID‐19

It is not yet known whether the pandemic has led to increased incidence of suicide in the general population (O'Connor et al., 2021; Pirkis et al., 2021). Those at increased risk of negative mental health during the pandemic include, those with pre‐existing mental health conditions, the elderly or very young people, women, minority groups, and those from socially disadvantaged backgrounds (Iob et al., 2020; Losada‐Baltar et al., 2021; O'Connor et al., 2021; Pierce et al., 2020; Ramiz et al., 2021). There is some evidence that the pandemic has brought upon an increase in suicidal thoughts (O'Connor et al., 2021; Sokoloff et al., 2021), which are strong predictors of suicide attempt (Klonsky et al., 2017; Mars et al., 2019; Nock et al., 2009; Nock et al., 2013). A longitudinal study from the United Kingdom (UK) suggests effects may be most pronounced in young adults (i.e., 18–29 years) (O'Connor et al., 2021). In the United States (US), a retrospective review of pediatric emergency department use during the early stages of the COVID‐19 pandemic identified a doubling of visits for suicidal ideation, suicide attempt, or self‐harm (Sokoloff et al., 2021). However, in a multi‐country study, others have found no evidence of a significant increase in suicide rates since the beginning of the pandemic, with the number of suicide deaths showing a significant decrease compared to the expected rate (Pirkis et al., 2021). This finding may be due to data being collected during the early stages of the pandemic, thereby concealing longer‐term effects (Appleby, 2021; Pirkis et al., 2021). Although declines in suicide during the early stages of the pandemic were reported in Japan, there was an increase in suicide rates during the second wave (Tanaka & Okamoto, 2021). Moreover, the largest increase in suicide was among women (37%) and young people aged below 20 years (49%).

## 3 SUICIDE RISK AND AUTISM

There is consistent evidence of increased suicide risk among the autistic population (Hirvikoski et al., 2016; Jokiranta‐Olkoniemi et al., 2021; Kirby et al., 2019; Kõlves et al., 2021; see Hedley & Uljarević, 2018 for a review). Increased risk of both suicide attempts and death by suicide ranges from three to nine times in autistic compared to non‐autistic populations (Hirvikoski et al., 2016; Kirby et al., 2019; Kõlves et al., 2021). There is emerging evidence that autistic traits or phenotype contribute to heightened suicide risk (Cassidy et al., 2018; Hedley et al., 2021; Stanley et al., 2021). Furthermore, in autistic populations, enhanced vulnerability to suicide is perhaps due to a high likelihood of co‐occurring mental health conditions (Jokiranta‐Olkoniemi et al., 2021). One of the most prevalent clinical conditions in this population is depression (lifetime rates may be as high as 49%) (Hudson et al., 2019; Lai et al., 2019; see Uljarević et al., 2020 for a review), which is strongly associated with suicidal thoughts and behavior (Franklin et al., 2017; Hedley, Uljarević, Wilmot, et al., 2018; Jokiranta‐Olkoniemi et al., 2021; Nock et al., 2009; Nock et al., 2013).

When considering psychological distress, it is equally important to consider protective factors and resilience (Keyes, 2005; Keyes et al., 2002; Teismann, Forkmann, et al., 2018). Positive mental health, or wellbeing, is an important construct that may protect against the negative mental health impacts of stressful events. For example, wellbeing has been found to act as a buffer against depression in employed autistic adults (Hedley et al., 2019). In non‐autistic samples, wellbeing moderates the association between depression and suicidal ideation (Teismann, Forkmann, et al., 2018), and is a protective factor against suicidal attempts in inpatients experiencing suicidal ideation (Teismann, Brailovskaia, et al., 2018).

## 4 COVID‐19 IMPACT IN AUSTRALIA

At the peak of the pandemic in 2020, Australia reported 721 new daily cases (July 31, 2020) and 59 deaths (September 25, 2020), reflecting a smaller impact than many countries. Nonetheless, as was the case around the world, Australians experienced significant disruptions to their daily lives and, by the end of 2020, Australia had reported 29,988 confirmed cases of COVID‐19 and 910 deaths (World Health Organization, 2021).

## 5 CURRENT STUDY

The present study examined potential associations between self‐reported COVID‐19 impact, personal wellbeing, depression and suicide risk factors assessed with the Suicide Behavior Questionnaire, Revised (SBQ‐R) (Osman et al., 2001) in autistic adults during the height of the second wave of the COVID‐19 pandemic in Australia (June to October, 2020) (World Health Organization, 2021). Based on the literature from both general and autistic populations, summarized above (e.g., Bal et al., 2021; Hedley et al., 2019; Hedley, Uljarević, Foley, et al., 2018; O'Connor et al., 2021; Teismann, Forkmann, et al., 2018), we hypothesized that: (a) wellbeing would be a protective factor and therefore negatively associated with COVID‐19 impact, depression and SBQ‐R risk score; (b) depression would be a risk factor and therefore positively associated with COVID‐19 impact and SBQ‐R risk score; and (c) COVID‐19 impact would significantly contribute to SBQ‐R risk score when controlling for personal wellbeing and depression. Age and gender effects were also investigated, predicting that COVID‐19 impact would be: (d) negatively associated with age; and (e) have a greater impact in women than men. Finally, participants' responses to COVID‐19 were explored using qualitative analysis.

## 6 METHOD

The study employed a mixed‐method survey design. Quantitative data were collected via an online survey, qualitative data were collected in response to a single (non‐obligatory) open question. Data were collected between October and December 2020.

### 6.1 Participants

Participants were 111 (women = 58.6%, men = 32.4%, non‐binary/not disclosed = 9.0%) English speaking adults aged 20 to 71 years (*M*_age_ = 42.45, SD *=* 13.03 years). Inclusion criteria included being aged 18 years or older and reporting a formal diagnosis of ASD by a qualified health professional. This was achieved by participants: (a) confirming verbally and in writing that they had received a formal diagnosis of autism and (b) providing details of their specific diagnosis (e.g., ASD and Asperger's Syndrome) and year of diagnosis. Participants identified at any stage of the study as having mental health or other safety concerns, which could place them at risk or cause undue distress were reviewed for inclusion.

### 6.2 Procedure

The study was approved by the La Trobe University Human Research Ethics Committee (HEC20235). All participants provided written informed consent. The study was advertised in an emailed newsletter sent to all participants from one of two Australian longitudinal studies on autism (see Arnold et al., 2019; Autism CRC, 2021 for further information). Study data were collected and managed using Research Electronic Data Capture (REDCap) electronic data capture tools hosted at La Trobe University (Harris et al., 2009; Harris et al., 2019). REDCap is a secure, web‐based application designed to support data capture for research studies, providing (1) an intuitive interface for validated data entry; (2) audit trails for tracking data manipulation and export procedures; (3) automated export procedures for seamless data downloads to common statistical packages; and (4) procedures for importing data from external sources. All participants were provided with links to mental health resources; those identified with any level of suicidal risk based on their responses to survey items were followed up according to the risk management protocol below. Participants were reimbursed AUD $100 in shopping vouchers upon completion of the study.

#### 6.2.1 Risk management protocol

A detailed risk assessment procedure based on an existing protocol (Byme et al., 2021) was developed for the study. Where any level of risk was identified, research staff contacted the participant to ascertain current risk level according to an algorithm (i.e., low, moderate, and high) and to ensure participant safety. The protocol included: (a) identification of available supports (e.g., family and health professionals); (b) formal referrals or provision of information about local services; (c) telephone or email checks by trained research staff of all people returning “low risk”; and (d) formal risk assessment by a licensed psychologist if risk level was classified as “moderate” or higher. As part of the informed consent process, participants provided contact details for an emergency contact and agreed to the study team contacting this person if there were concerns for the participant's safety. Where there was concern that the participant was at imminent risk of self‐harm or suicide, the protocol was for the research team to contact emergency services, although this was not required.

### 6.3 Measures

#### 6.3.1 Demographics

Participants reported basic demographic and diagnostic information.

#### 6.3.2 COVID‐19 impact

The COVID‐19 Impact Scale (CIS) (Stoddard et al., 2021; Stoddard & Kaufman, 2020) is a 12‐item self‐report assessment of the impact of the COVID‐19 pandemic on a person's life. On items one to eight, respondents rate, on a four‐point Likert scale ranging from 0 (no change) to 3 (severe), how much the pandemic has changed their life in the following areas: routines, family income/employment, food access, medical health care access, mental health treatment access, access to extended family and non‐family social supports, stress, and family discord. When summed, these items provide a reliable (*α =* 0.64–0.75) and unitary scale of COVID‐19 impact (range: 0–24) (Stoddard et al., 2021). Items nine to 11 assess whether the participant, family member, or friends had contracted COVID‐19. As these items do not contribute to the CIS impact scale they are reported separately. The final item (i.e., 12) is an open‐ended question. In the present study, internal consistency (using McDonald's Omega) for the CIS eight items was good, *ω* = 0.819.

#### 6.3.3 Wellbeing

Wellbeing was assessed with the Personal Wellbeing Index, Adult (PWI‐A), fifth edition (International Wellbeing Group, 2013). The PWI‐A assesses satisfaction with life across seven domains: standard of living, health, life achievement, personal relationships, personal safety, community connectedness, and future security using an 11‐point end‐defined response scale ranging from 0 (no satisfaction at all) to 10 (completely satisfied). A total score is summed and averaged prior to being converted to a standardized scale (0–100). The PWI‐A has been validated in autistic adults demonstrating good internal consistency (*α =* 0.87) (Thorpe, 2018). In the present study internal consistency was good, *ω* = 0.859.

#### 6.3.4 Depression

Depressive symptoms were assessed with the Patient Health Questionnaire, eight‐item (PHQ‐8) (Kroenke et al., 2009; Kroenke & Spitzer, 2002), which is effective in screening for major and subthreshold depressive symptoms in clinical and general population samples (Kroenke et al., 2009; Shin et al., 2019). The full PHQ‐9, which includes one additional question addressing self‐harm/suicide risk, has been validated in autistic adults (*α =* 0.91) (Arnold et al., 2020). For the present study, PHQ‐8 was used to avoid conflation by inclusion of the suicidality question with the SBQ‐R. Respondents rate how much they are affected by problems on a four‐point scale ranging from 0 (not at all) to 3 (nearly every day). Total scores range from 0 to 24. Higher scores indicate greater depressive symptoms. In the present study internal consistency was good, *ω* = 0.868.

#### 6.3.5 Suicide risk factors

Suicide risk factors were assessed with the SBQ‐R (Osman et al., 2001), a four‐item self‐report questionnaire designed to identify specific risk factors for suicide (e.g., threat of suicide attempt, self‐reported likelihood of future suicidal behavior). Scores are summed across items with a total range from 3 to 18. Higher scores indicate greater risk and a score equal to or greater than seven provides good sensitivity and specificity for suicide behavior. Autistic adults tend to score higher on the SBQ‐R, and are more likely to be in the clinical range than adults from the general population (Cassidy et al., 2018). In the present study internal consistency was acceptable and comparable to previous research (e.g., Cronbach's *α* = 0.76; Cassidy et al., 2018), *ω* = 0.769.

### 6.4 Data analysis

#### 6.4.1 Quantitative data

No more than 1% (*M =* 0.11, SD *=* 0.32) of data were missing from any questionnaire overall. Little's (1988) MCAR was not significant, *p =* 0.498. An inspection of missing data revealed one participant was missing one item on the PWI‐A, and another was missing two items on the PWI‐A. Data were imputed for these three missing items using the mean PWI‐A score for the participant. There were no missing data for any of the other variables and no extreme outliers were identified. Visual and statistical analysis of distributions revealed a positive skew of CIS scores (*z*_skewness_ = 3.14, *p <* 0.01) which was not corrected by transformation. No other distributions were significantly skewed (all *z*_skewness_ < 0.98, *p >* 0.05). Bootstrapping with 5000 resamples was used to correct for non‐normality of data (Bishara & Hittner, 2012; see also Field, 2018; Howell, 2013). Bootstrapping corrected for the skewness in the CIS (bootstrapped *z*_skewness_ = 0.425, *p >* 0.05). Significance was interpreted by referring to both Bonferroni corrected *p‐*values and bootstrapped 95% confidence intervals (BCa 95% CI) (Efron & Tibshirani, 1993; Tabachnick & Fidell, 2007). Cohen's *d* was used to interpret effect size (Cohen, 1988).

Correlational analyses were used to initially explore associations between study variables, including partial correlations controlling for age. Next, between‐group comparisons were used to examine gender differences on each measure and correlations between measures were examined for women and men separately. Fisher's transformation was used to compare whether there were statistically significant differences in the strength of the correlations between women and men. Finally, multiple linear regression with all theoretical variables (i.e., COVID‐19 impact, personal wellbeing, depression) entered at once was used to identify the factors contributing to SBQ‐R suicide risk score.

#### 6.4.2 Qualitative data

To gain a more nuanced appreciation of how participant's had been impacted by COVID‐19, we examined qualitative responses provided to the open‐ended question (i.e., item 12) of the CIS (Stoddard & Kaufman, 2020; Stoddard et al., 2021, May 24), *‘Please tell us about any other ways the coronavirus pandemic has impacted your life’*. Responses were coded thematically using a deductive approach (Braun & Clarke, 2006) into one of three categories: (a) no change/neutral impact; (b) mild/positive impact; and (c) moderate or severe/negative impact. A single‐blind procedure was followed whereby the first and second coders (one autistic and the other not autistic) independently placed the participant response into one of the three categories. Where there was disagreement, the two coders discussed and agreed upon placement of the response. Before placement of the response was agreed upon, inter‐rater reliability was very high, *κ = 0*.96.

## 7 RESULTS

### 7.1 Demographics

The combined pool of potential participants who had completed wave one of the longitudinal studies was *n =* 467. Overall, there were 135 expressions of interest for the current study, representing approximately 29% of the potential participant pool. Of those who consented into the study, eight participants were excluded for either a) not reporting a formal autism diagnosis (*n =* 7; six women, one non‐binary person; *M*_age_ = 53.29, SD = 12.89) or b) ethical and safety concerns (*n =* 1). The final sample consisted of 103 adults (women = 57.3%, men = 34%, non‐binary/not disclosed = 8.8%; *M*_age_ = 41.71, SD *=* 12.83 years, range = 20–71). Participant demographics and diagnostic information are provided in Table 1. Almost 85% (*n =* 87) had completed a post‐secondary qualification, 78% (*n =* 80) reported living with someone else, and 53% (*n =* 55) had some employment. Co‐occurring diagnoses of anxiety or depression were reported by 70% (*n =* 73, *n =* 71, respectively) of participants.

**TABLE 1:** Participant characteristics

Variable	Label	*n* (%)
Birth country	Australia	82 (79.6%)
	Other	21 (20.4%)
Language at home	English	97 (94.2%)
	Other	6 (5.8%)
Education	Secondary school	16 (15.5%)
	Certificate or diploma	26 (25.2%)
	Bachelor's degree	33 (32%)
	Master's degree	23 (22.3%)
	PhD	5 (4.9%)
Living	Spouse or partner	40 (38.8%)
	Housemates	13 (12.6%)
	Alone	23 (22.3%)
	Parents or another relative	20 (19.4%)
	Other	7 (6.8%)
Relationship status^a^	Single	43 (41.7%)
	Partnered	28 (27.2%)
	Same sex relationship	7 (6.8%)
	Married/engaged	24 (23.3%)
	Separated/divorced	11 (10.7%)
	Other	6 (5.8%)
Employment	Full‐time (35+ h/week)	23 (22.3%)
	Part‐time	32 (31.1%)
	Seeking employment	13 (12.6%)
	Not seeking employment	24 (23.3%)
	Retired	11 (10.7%)
Autism diagnostic type	Autism spectrum disorder	53 (51.5%)
	Autistic disorder	1 (1%)
	Asperger's syndrome	43 (41.7%)
	High functioning autism	5 (4.9%)
	PDD‐NOS^b^	1 (1%)
Other diagnoses	Anxiety	73 (70.9%)
	Depression	71 (68.9%)
	Developmental delay	1 (1%)
	Intellectual disability	1 (1%)
	Speech/language impairment	4 (3.9%)
	ADHD	35 (34%)
	Asthma	25 (24.3%)
	Allergy	25 (24.3%)
	Hearing impairment/deaf	5 (4.9%)
	Orthopedic impairment	7 (6.8%)
	Seizure disorder/epilepsy	3 (2.9%)
	Visual impairment	9 (8.7%)
	Other diagnosis	33 (32%)
	None	7 (6.8%)
	Prefer not to answer	1 (1%)

^a^ Total exceeds 100% due to multiple selections possible.

^b^ Pervasive developmental disorder not otherwise specified.

Table 2 provides a summary of participant distribution across the Australian states and territories, as well as comparison population data (from Australian Bureau of Statistics, 2021). Compared to expected population proportions, New South Wales (NSW) was underrepresented, and the Australian Capital Territory (ACT) was overrepresented (refer to Table 2). Differences in CIS scores were examined between states and territories. Participants from Victoria (VIC) reported the highest overall COVID‐19 impact scores (*M =* 9.94, SD *=* 5.25). A one‐way analysis of variance (ANOVA) based on 5000 bootstrap samples indicated non‐significant differences for COVID‐19 impact scores between the states and territories, *F*(6,95) = 2.190, *p =* 0.051, *η* ^2^ = 0.12. Due to small sample size from several states and territories and the overall significance level being greater than 0.05, these differences were not considered further. All data and Bonferroni corrected multiple comparisons are provided in Table S1.

**TABLE 2:** Participant COVID‐19 impact score (CIS) and participant distribution by state or territory

	CIS		Population (%)^a^	Difference from expected, *p* value
State/territory	*M* (SD)	Study *n* (%)		
VIC^c^	9.94 (5.248)	32 (31.1%)	26.0%	0.140
NSW^c,d^	7.95 (4.572)	22 (21.4%)	31.8%	**0.018** ^b^
ACT^c,d,e^	8.11 (3.219)	9 (8.7%)	1.7%	**0.009** ^b^
QLD^d,e,f^	6.59 (4.925)	22 (21.4%)	20.2%	0.750
WA^d,e,f^	6.22 (4.549)	9 (8.7%)	10.4%	0.560
SA^f^	5.50 (1.732)	4 (3.9%)	6.9%	0.210
TAS^f^	3.75 (2.872)	4 (3.9%)	2.1%	0.190
NT^−^	–	0 (0%)	1.0%	0.290
Not reported	–	1 (1%)	n/a	–

*Note*: Results that are statistically significant following Bonferroni corrections are marked in bold.

^a^ Source Australian Bureau of Statistics, 2021.

^b^ Sample proportion differed significantly from expected Australian population.

^c‐f^ CIS means with the same superscripts do not differ significantly based on 5000 samples bootstrapped Bonferroni corrected multiple comparisons (refer to Table S1 for statistics).

#### 7.1.1 Personal exposure to COVID‐19

Personal exposure to COVID‐19 was assessed with items nine to 11 on the CIS. One participant reported a COVID‐19 diagnosis with mild symptoms. A further eight participants reported knowing a friend or extended family member who had been diagnosed with COVID‐19, with one COVID‐19 associated death reported in this group.

### 7.2 Quantitative analyses

#### 7.2.1 Correlations and age effects

Table 3 provides descriptive statistics and correlations between the main study variables. Patterns of associations between variables for full and partial correlations controlling for age were similar with only minor differences in effect size. As age was not significantly correlated with any of the primary study variables, it was not considered further in analyses. Although bootstrapped correlations suggested small associations for COVID‐19 impact with personal wellbeing, *r =* −0.224, *p =* 0.023, 95% BCa CI [−0.409, −0.016], and depression, *r =* 0.268, *p =* 0.006, [0.056, 0.445], these associations were not statistically significant when applying Bonferroni corrected *p* values. Both personal wellbeing (negatively), *r =* −0.590, *p <* 0.001, [−0.724, −0.427], and depressive symptoms (positively), *r =* 0.439, *p <* 0.001, [0.273, 0.587], were significantly correlated with suicide risk on the SBQ‐R. However, the association between COVID‐19 impact and SBQ‐R suicide risk was small and not statistically significant.

**TABLE 3:** Study variables (*M*, SD, range) with Bonferroni corrected Pearson's bootstrapped^a^ correlations (upper panel) and partial correlations (lower panel) controlling for age, *n =* 103

Variable	*M*	SD	Range	2.	3.	4.	5.
1. Age	41.71	12.83	20.89–70.92	−0.123 [−0.287,0.062]	−0.061 [−0.226,0.119]	−0.094 [−0.270,0.083]	−0.041 [−0.214,0.137]
2. CIS	7.87	4.82	0–23	–	−0.224 [−0.409,−0.016]	0.268 [0.066,0.453]	0.081 [−0.116,0.267]
3. PWI‐A	52.03	20.61	2.86–98.57	−0.234 [−0.432,‐0.018]	–	−0.682* **[−0.772,‐0.564]**	−0.586* **[−0.718,‐0.430]**
4. PHQ‐8	10.45	6.15	0–24	0.260 [0.056,0.445]	−0.692* **[−0.783,‐0.574]**	–	0.441* **[0.270,0.591]**
5. SBQ‐R	10.04	3.76	3–18	0.076 [−0.123,0.267]	−0.590* **[−0.724,‐0.427]**	0.439* **[0.273,0.587]**	–

*Note*: *p < 0.001. Results that are statistically significant following Bonferroni corrections are marked in bold.

^a^ 5000 sample, 95% bias‐corrected and accelerated (BCa).

#### 7.2.2 Gender

To explore possible differences between women and men^1^ we first examined mean scores on the study variables and conducted between‐group comparisons (see Table 4). Overall, scores for women and men were similar, and not significantly different across measures. There were small effects for differences between women and men on COVID‐19 impact (*d =* 0.35) and SBQ‐R (*d =* 0.26), and these differences were not statistically significant (*p* values ≥ 0.107).

**TABLE 4:** Descriptive (*M*, SD) and bootstrapped between‐group comparisons for study variables by gender (females: *n =* 59; males: *n =* 35)

Variable	Female		Male			Between group comparisons		
	*M*	SD	*M*	SD	*t*(92)	*p* value	BCa 95% CI^a^	Cohen's *d* [95% CI]
Age	41.69	11.90	43.82	14.79	0.727^b^	0.47	−3.78,7.96	0.16 [−0.26,0.58]
CIS	8.29	4.71	6.66	4.66	−1.630	0.11	−3.58,0.374	0.35 [−0.07,0.77]
PWI‐A	51.63	21.61	53.39	20.31	0.389	0.70	−6.79,10.48	0.08 [−0.34,0.50]
PHQ‐8	10.44	6.19	10.63	5.92	0.145	0.89	−2.43,2.79	0.03 [−0.39,0.45]
SBQ‐R	10.25	4.03	9.26	3.35	−1.233	0.22	−2.59,0.569	0.26 [−0.16,0.68]

^a^ 5000 sample, 95% bias‐corrected and accelerated (BCa).

^b^ Results reported for equal variances not assumed due to Levene's Test for equality of variances <0.05, df *=* 59.925.

Table 5 highlights the pattern of associations between study variables for women and men. For both women and men, the negative associations between personal wellbeing and both depression and SBQ‐R suicide risk were significant. Additionally, for women, SBQ‐R suicide risk was positively associated with depression. Notably in the present study, COVID‐19 impact scores showed very weak associations with SBQ‐R scores in both women *r =* 0.01, *p =* 0.942, 95% BCa CI [−0.267,0.282], and men, *r =* 0.13, *p =* 0.455, [−0.204,0.411].

**TABLE 5:** Pearson's bootstrapped^a^ correlations for females (upper panel; *n =* 59) and males (lower panel; *n =* 35)

Variable	1.	2.	3.	4.
1. CIS	–	−0.182 [−0.459,0.109]	0.326 [0.065,0.543]	0.010 [−0.259,0.271]
2. PWI‐A	−0.169 [−0.444,0.160]	–	−0.788* **[−0.859,‐0.694]** ^b^	−0.637* **[−0.787,−0.441]**
3. PHQ‐8	0.187 [−0.141,0.485]	−0.528* **[−0.736,‐0.242]** ^b^	–	0.589* **[0.350,0.766]** ^c^
4. SBQ‐R	0.130 [−0.211,0.445]	**−0.501** * **[−0.727,−0.225]**	0.237 [−0.088,0.520]^b^	–

*Note*: *p < 0.002. Results that are statistically significant following Bonferroni corrections are marked in bold.

^a^ 5000 sample, 95% bias‐corrected and accelerated (BCa).

^b^ Fisher's *r* to *z* transformations revealed that the strength of the correlations differed significantly between males and females.

*p < 0.002.

Fisher's transformation (Salkind, 2007) was used to compare for statistically significant differences in the strength of the correlations between women and men. No statistically significant differences between women and men were identified for the associations between COVID‐19 impact and personal wellbeing (*z =* −0.061, *p =* 0.476), depression (*z =* 0.673, *p =* 0.250), or SBQ‐R risk score (*z =* −0.545, *p =* 0.293), or between personal wellbeing and SBQ‐R risk score (*z =* −0.914, *p =* 0.180). However, the strength of correlations suggested a difference between genders for personal wellbeing and depression (*z =* −2.16, *p =* 0.015), and depression and SBQ‐R risk score (*z =* 1.961, *p =* 0.025). In both cases, the strength of the association was stronger in women than men.

#### 7.2.3 Regression analyses

Table 6 presents the results of the linear regression model examining COVID‐19 impact, personal wellbeing and depression as predictors of SBQ‐R risk score. The full model accounted for 35.1% of variance in SBQ‐R scores, *F*(3, 99) = 17.82, *p <* 0.001. Personal wellbeing emerged as a significant predictor of SBQ‐R scores, with the *b*‐weight revealing that for each unit increase in personal wellbeing, SBQ‐R scores decreased by −0.098 units, making the largest significant contribution (*β =* −0.539) to SBQ‐R scores overall. Neither COVID‐19 impact (*β =* −0.064) nor depression (*β =* 0.091) emerged as significant predictors of SBQ‐R risk score in the model. A second model was conducted to explore gender effects, with gender coded as a binary variable (man = 0, woman = 1). Gender did not make a significant contribution to the model; overall results were similar to the first model. Results for the second model are provided in Table S2.

**TABLE 6:** Linear regression model of predictors of SBQ‐R suicide risk scores

	*b*	SEB^a^	*β*	*p* value	BCa 95% CI
Constant	14.97	1.639	–	<0.001	**11.85, 18.40**
COVID‐19	−0.050	0.066	−0.064	0.448	−0.180. 0.065
Personal Wellbeing	−0.098	0.019	−0.539	<0.001	**−0.133, −0.063**
Depression	0.056	0.067	0.091	0.399	−0.078, 0.187

*Note: R* ^2^ = 0.351, *F*(3, 99) = 17.82, *p <* 0.001. Results that are statistically significant following Bonferroni corrections are marked in bold. BCa 95% confidence intervals that do not cross zero are bolded. *p* values and 95% bias corrected and accelerated confidence intervals and standard errors based on 5000 bootstrap samples.

^a^ SEB: the standard error for the unstandardized beta.

### 7.3 Qualitative analysis

Responses to the open‐ended question from the CIS were received from *n* = 72 participants (women = 64%; men = 30.5%; non‐binary/not disclosed = 5.5%; *M*_age_ = 43.06, SD *=* 13.06). The following exemplar quotes are illustrative of the responses received, and have been coded into three overarching themes: No Change/Neutral Impact; Mild/Positive Impact; Moderate or Severe/Negative Impact. To assist in the protection of participants' identity, we report participants' age as falling within a 10‐year range.

#### 7.3.1 No change/neutral impact

Few (18%; *n* = 13; women = 76.9%) participants described either a neutral or balanced viewpoint concerning the impact of COVID‐19. Participant responses coded to this category stated that COVID‐19 has not impacted their life, or they presented both negative and positive impacts of COVID‐19. For example, this participant reported that COVID‐19 had not affected her. However, it is notable that this was primarily due to having limited contact with family and friends under usual circumstances:

“We are such a hermit family that it didn't bother us. We have limited family, most of whom wait for us to contact them […]. We have little to no friends.” [woman, 40–49 years]

Another participant commented that she enjoyed having remote meetings for work, but she also missed the contact with others:

“There has been good as well as bad. Good is having Zoom meetings which make my non‐profit job easier, the bad is no touch and I like touch with some people.” [woman, 60–69 years]

Finally, one participant stated that they were spending less money because of COVID‐19, viewing this as a positive outcome, but he also felt disappointed with humanity because of the pandemic:

“Spending less money. More disappointed in humanity in general.” [man, 30–39 years]

#### 7.3.2 Mild/positive impact

A mild, or rather positive impact on participant's wellbeing was reported by 22% (*n* = 16; women = 62.5%) of participants. In their qualitative response they described COVID‐19 as having only a positive impact on them. Reasons provided were related to reduced stress, most frequently resulting from reduced social obligations and pressures. Circumstances for reduced stress included participants: being able to work or study from home; fewer crowded areas when required to leave their home, and; being in a better financial position owing to increases in Government welfare assistance or obtaining employment. For example, one participant commented on the positive impact working from home had made on him:

“I have been working from home, and this has really helped me both in productivity and in mental health. My wife has also commented on my mental health, and is happy having me work from home.” [man, 50–59 years]

Another participant also identified positive impacts due to having fewer social demands:

“Due to the lockdown and social distancing, I can enjoy a bit more to go to the public without worrying the crowd. Also, I do not need to feel missing out on social occasions or opportunities to meet people. As no one is socialising or meeting new people. A lot less social pressure in general.” [man, 20–29 years]

This participant also noted a benefit associated with fewer social demands:

“Change for the better. For a short while, I no longer had to make excuses to stay home, no longer had to justify why I didn't want to go out.” [woman, 30–39 years]

#### 7.3.3 Moderate or severe/negative impact

For the majority (60%; *n* = 43; women = 60.5%) of participants, the second COVID‐19 wave resulted in increased stress and exacerbated pre‐held or existing mental health conditions. These respondents only reported negative impacts and thus were coded as having moderate or severe/negative impact. Reasons for stating that COVID‐19 had a negative impact were due to: uncertainty, unpredictability, or loss of control/freedom; loss of social/emotional supports or social opportunities; unemployment including employment uncertainty and reduced employment; increased workload or work stress; sensory issues related to mask wearing; concerns about contracting or becoming a COVID‐19 vector; worry over the health and behavior of others; unavailability and limited availability of services, shops or needed items, and; home schooling children as well as friction between family/home interpersonal relationships. For example, in the following statement the participant, who was experiencing distress associated with her mental health, describes being turned away from hospital:

“[I was] turned away from hospital, as told mental health could be treated by phone instead.” [woman, 30–39 years]

In this example, the participant describes significant impacts on her mental health and friendships that she associated with COVID‐19:

“I've backslid so far with depression and anxiety that it feels impossible now. I lost a lot of friends […] I feel like the pandemic completely derailed everything concerning my mental health and every effort I have taken to improve my life. I feel incredibly isolated.” [woman, 40–49 years]

## 8 DISCUSSION

The present study examined potential associations between self‐reported COVID‐19 impact and suicide risk factors assessed with the SBQ‐R in Australian autistic adults. COVID‐19 impact showed a small but not statistically significant association with poorer personal wellbeing and higher depressive symptoms, and was not significantly associated with suicide risk factors; the regression model indicated that the only significant predictor of SBQ‐R risk score was personal wellbeing. Thus, our hypothesis predicting an association between COVID‐19 impact and SBQ‐R suicide risk was not supported. Consistent with others, our findings suggest associations between wellbeing and mental health (Hedley et al., 2019; Keyes, 2005; Keyes et al., 2002), and wellbeing and suicide risk (Teismann, Brailovskaia, et al., 2018; Teismann, Forkmann, et al., 2018). Our finding that depression was not a significant predictor of SBQ‐R risk when controlling for personal wellbeing is somewhat inconsistent with the literature (Franklin et al., 2017; Hedley, Uljarević, Wilmot, et al., 2018; Jokiranta‐Olkoniemi et al., 2021; Nock et al., 2009). However, this finding is consistent with models suggesting a degree of independence between depression and suicide risk, and a role for positive mental health as a protective factor in the path from suicidal ideation to suicidal behavior (e.g., dual factor model; Teismann, Brailovskaia, et al., 2018). We found no significant association between participant age and any of the primary study variables.

While a weak and not significant negative association between COVID‐19 impact and wellbeing is possibly suggestive of greater negative than positive impact overall, COVID‐19 in and of itself was not a predictor of increased suicide risk score. This is despite the pandemic's potential impact on autistic people specifically due to pre‐existing heightened risk of suicide (Cassidy et al., 2018; Hedley & Uljarević, 2018), or broad risk factors such as reduced social support which is typically associated with suicide risk (Hedley et al., 2017; Hedley, Uljarević, Foley, et al., 2018; Hedley, Uljarević, Wilmot, et al., 2018).

Many of the current sample were autistic females and therefore at higher risk of negative mental health outcomes associated with COVID‐19. First, compared to non‐autistic females, autistic females are at increased risk of suicide (Hirvikoski et al., 2016; Kirby et al., 2019). Second, greater psychological distress associated with COVID‐19 has been observed in autistic women (Bal et al., 2021). However, the models for SBQ‐R suicide risk were similar when examining women and men separately, although the strength of the associations between wellbeing and depression, as well as between depression and SBQ‐R risk score, were somewhat stronger in women than in men. Overall, our findings did not support our hypothesis that autistic women would be more affected by the impact of the pandemic than autistic men.

Our analysis of responses to the open‐ended question in the CIS offers a more nuanced appreciation of the association between COVID‐19 impacts and mental health. Consistent with others (Eshraghi et al., 2020; Oomen et al., 2021), COVID‐19 had both positive and negative impacts on participant wellbeing. It is possible that the positive impacts of COVID‐19 will have served as a protective factor against the negative impacts. Similarly, hope, which could be considered a feature of positive mental health, has been found to be protective against psychological distress associated with COVID‐19 in autistic people (Bal et al., 2021). It may also be that circumstances brought upon by COVID‐19 (e.g., reduced social pressure) played to autistic people's preferences or strengths making them resilient. However, this hypothesis is very preliminary and warrants further detailed exploration.

Gender distribution for each category was similar to the overall distribution (i.e., 64% women) for Mild/Positive Impact and Moderate or Severe/Negative Impact (i.e., 62.5%, 60.5% women, respectively) categories. A slightly higher portion of responses from women (76.9%) than men (23.1%) were coded in the No Change/Neutral Impact category; however, this result is difficult to interpret given a relatively low number of responses (*n =* 13) coded to this category overall. Thus, no clear gender differences were observed for the qualitative results.

### 8.1 Limitations

Our findings are limited by the use of a cross‐sectional survey design. Second, relative to many other countries, the pandemic impact in Australia could be considered mild in terms of overall death and infection rates, which could temper the potential association between COVID‐19 and suicide risk factors thereby limiting the generalizability of findings to countries with higher rates of infection or more severe impacts. It is worth noting that approximately one third of participants (31%) were from the state of Victoria, which had in place the longest and most restrictive stay at home orders in Australia. Leading up to December 2020, Victoria had been in Stage 4 lockdown (stay at home order, restricted access to public spaces, no interaction with people from other households, compulsory masks both indoors and outdoors) for approximately 8 months. Other states had similar restrictions imposed for reduced periods of time in response to widespread or local outbreaks. Nonetheless, the nature of ‘impact’ as measured by the CIS scale emphasizes the social and familial impact of COVID‐19 (e.g., mental health treatment access, social supports, stress). Thus, with regards to potential risk factors for increased mental health difficulties (e.g., stress) (Cooke et al., 2020; Corbett et al., 2021; Salari et al., 2020) and suicide (e.g., reduced social support) (Holmes et al., 2020; O'Connor et al., 2021), the potential for impact on mental health and wellbeing in Australia was substantial and borne out by research (Newby et al., 2020). We must also acknowledge the potential for bias in our sample, which was only advertised to autistic people who had previously participated in one of two longitudinal autism studies. Coupled with an initial response rate of 29% from this sample, generalization of results to the broader autistic population, as well as those with higher needs or intellectual disability, is limited. Although similar in age and gender distribution to the longitudinal sample (Arnold et al., 2019), the study sample is not reflective of the male‐to‐female ratio of autism, which is about 3:1 (Loomes et al., 2017). Given the small number of males relative to females, our interpretation of gender results should be interpreted cautiously. Further, many participants (97%) in the study required follow‐up due to reporting at least some level of suicide risk,^2^ suggesting bias toward those with pre‐existing mental health concerns. Last, it is important to note that the SBQ‐R was not designed specifically for use with autistic people. Although a version of this instrument has now been validated for use in autistic adults (Cassidy et al., 2021), it was not available when the present study was conceived.

## 9 CONCLUSION

The COVID‐19 pandemic represents a massive upheaval of life as normal, with the potential to lead to greater mental health challenges and increased suicide risk factors. It is important to consider potential impacts on the most vulnerable and those known to have an existing heightened risk for suicide. Therefore, our study aim was to examine whether COVID‐19 impact was associated with suicide risk factors among Australian autistic adults. While COVID‐19 impact showed a small association with increased depressive symptoms and reduced personal wellbeing, it was not directly associated with SBQ‐R suicide risk when controlling for these factors. Positive wellbeing was found to be associated with lower SBQ‐R suicide risk, even during a period of significant stress and challenge. Further research is needed to more directly investigate the possible protective nature of wellbeing on suicide risk among autistic people.

## CONFLICT OF INTEREST

Darren Hedley is supported by a Suicide Prevention Australia National Suicide Prevention Research fellowship. Mirko Uljarević is supported by a Discovery Early Career Researcher Award from the Australian Research Council (DE180100632). Jo Robinson is supported by a National Health and Medical Research Council (NHMRC) Career Development Fellowship (APP1142348) and a University of Melbourne Dame Kate Campbell Fellowship. The authors declare no actual or potential conflict of interest.

## AUTHOR CONTRIBUTIONS

Darren Hedley, Mirko Uljarević, and Mark A. Stokes conceived of and designed study. Data collection was managed by Darren Hedley with assistance from Kathleen Denney and Ensu Sahin. Darren Hedley completed the literature review, quantitative data analysis and interpretation. Susan M. Hayward, Kathleen Denney and Darren Hedley analyzed and wrote up the qualitative data. Darren Hedley, Susan M. Hayward and Kathleen Denney wrote the first draft. All authors contributed to, reviewed and approved subsequent and final versions of the manuscript.

10

## ETHICS STATEMENT

The research was approved by La Trobe University Human Research Ethics Committee HEC20235. All procedures performed in studies involving human participants were in accordance with the ethical standards of the institutional and/or national research committee and with the 1964 Helsinki declaration and its later amendments or comparable ethical standards. Informed consent was obtained from participants after the nature of the study was explained.

## Supporting information


**Table S1** Bootstrapped Bonferroni corrected multiple comparisons for COVID‐19 impact between Australian states and territories

**Table S2** Linear regression model of predictors of SBQ‐R suicide risk scores with gender (binary variable) entered into the model (*n =* 94)

## Data Availability

Requests for access to the data sample should be directed to Darren Hedley, PhD, Olga Tennison Autism Research Centre, School of Psychology and Public Health, La Trobe University, Melbourne 3086, VIC, Australia; e‐mail: d.hedley@latrobe.edu.au.

## Footnotes

^1^
Non‐binary participants were not included given the small number.

^2^
Three (2.9%) participants reported no suicide risk on any measure (i.e., no history of suicidal ideation or behavior). Following review by senior research staff (DH, MS) following the guidelines in the risk management protocol, 87 (84.5%) participants were assessed as having low risk of suicide and 13 (12.6%) were assessed as being at moderate risk.

## References

[aur2614-bib-0001] Appleby, L. (2021). What has been the effect of covid‐19 on suicide rates? The BMJ, 372, n834. 10.1136/bmj.n834 33782026

[aur2614-bib-0002] Arnold, S. , Foley, K.‐R. , Hwang, Y. I. , Richdale, A. L. , Uljarevic, M. , Lawson, L. P. , Cai, R. Y. , Falkmer, T. , Falkmer, M. , Lennox, N. G. , Urbanowicz, A. , & Trollor, J. (2019). Cohort profile: The Australian longitudinal study of adults with autism (ALSAA). BMJ Open, 9(12), e030798. 10.1136/bmjopen-2019-030798 PMC6924702 31806608

[aur2614-bib-0003] Arnold, S. R. C. , Uljarević, M. , Hwang, Y. I. , Richdale, A. L. , Trollor, J. N. , & Lawson, L. P. (2020). Psychometric properties of the patient health questionaire‐9 (PHQ‐9) in autistic adults. Journal of Autism and Developmental Disorders, 50(6), 2217–2225. 10.1007/s10803-019-03947-9 30847710

[aur2614-bib-0004] Australian Bureau of Statistics . (2021). *National, State and Territory Population, September 2020*. Commonwealth of Australia. https://www.abs.gov.au/statistics/people/population/national-state-and-territory-population/sep-2020

[aur2614-bib-0005] Autism CRC . (2021). *Snapshot profiles from the Study of Australian School Leavers with Autism (SASLA)*. Autism CRC. https://www.autismcrc.com.au/knowledge-centre/resource/snapshot-profiles-study-australian-school-leavers-autism-sasla

[aur2614-bib-0006] Bal, V. H. , Wilkinson, E. , White, L. C. , Law, J. K. , Feliciano, P. , & Chung, W. K. (2021). Early pandemic experiences of autistic adults: Predictors of psychological distress. Autism Research, 14(6), 1209–1219. 10.1002/aur.2480 33559334 PMC8014774

[aur2614-bib-0007] Baweja, R. , Brown, S. L. , Edwards, E. M. , & Murray, M. J. (2021). COVID‐19 pandemic and impact on patients with autism spectrum disorder. Journal of Autism and Developmental Disorders. Advance online publication. 10.1007/s10803-021-04950-9 PMC7943706 33689088

[aur2614-bib-0008] Bishara, A. J. , & Hittner, J. B. (2012). Testing the significance of a correlation with nonnormal data: Comparison of Pearson, Spearman, transformation, and resampling approaches. Psychological Methods, 17(3), 399–417. 10.1037/a0028087 22563845

[aur2614-bib-0009] Braun, V. , & Clarke, V. (2006). Using thematic analysis in psychology. Qualitative Research in Psychology, 3(2), 77–101. 10.1191/1478088706qp063oa

[aur2614-bib-0010] Brooks, S. K. , Webster, R. K. , Smith, L. E. , Woodland, L. , Wessely, S. , Greenberg, N. , & Rubin, G. J. (2020). The psychological impact of quarantine and how to reduce it: Rapid review of the evidence. The Lancet, 395(10227), 10912–10920. 10.1016/S0140-6736(20)30460-8 PMC7158942 32112714

[aur2614-bib-0011] Byme, S. J. , Lamblin, M. , Pirkis, J. , Mihalopoulos, C. , Spittal, M. J. , Rice, S. , Hetrick, S. , Hamilton, M. , Yuen, H. P. , Lee, Y. Y. , Boland, A. , & Robinson, J. (2021). Study protocol for the Multimodal Approach to Preventing Suicide in Schools (MAPSS) project: A regionally‐based trial of an integrated response to suicide risk among secondary school students. Research Square. 10.21203/rs.3.rs-518233/v1 PMC8889397 35236397

[aur2614-bib-0012] Cassidy, S. , Bradley, L. , Cogger‐Ward, H. , & Rodgers, J. (2021). Development and validation of the suicidal behaviours questionnaire—Autism spectrum conditions in a community sample of autistic, possibly autistic and non‐autistic adults. Molecular Autism, 12, 46. 10.1186/s13229-021-00449-3 34154642 PMC8218414

[aur2614-bib-0013] Cassidy, S. , Bradley, L. , Shaw, R. , & Baron‐Cohen, S. (2018). Risk markers for suicidality in autistic adults. Molecular Autism, 9, 42. 10.1186/s13229-018-0226-4 30083306 PMC6069847

[aur2614-bib-0014] Cohen, J. (1988). Statistical power analysis for the behavioral sciences (2nd ed.). Lawrence Erlbaum Associates.

[aur2614-bib-0015] Cooke, J. E. , Eirich, R. , Racine, N. , & Madigan, S. (2020). Prevalence of posttraumatic and general psychological stress during COVID‐19: A rapid review and meta‐analysis. Psychiatry Research, 292, 113347. 10.1016/j.psychres.2020.113347 32763477 PMC7392847

[aur2614-bib-0016] Corbett, B. A. , Muscatello, R. A. , Klemencic, M. E. , & Schwartzman, J. M. (2021). The impact of COVID‐19 on stress, anxiety, and coping in youth with and without autism and their parents. Autism Research. 10.1002/aur.2521 PMC8237027 33913261

[aur2614-bib-0017] Efron, B. , & Tibshirani, R. (1993). An introduction to the bootstrap. Chapman & Hall/CRC.

[aur2614-bib-0018] Eshraghi, A. A. , Li, C. , Alessandri, M. , Messinger, D. S. , Eshraghi, R. S. , Mittal, R. , & Armstrong, F. D. (2020). COVID‐19: Overcoming the challenges faced by individuals with autism and their families. The Lancet Psychiatry, 7(6), 481–483. 10.1016/S2215-0366(20)30197-8 PMC7239613 32445682

[aur2614-bib-0019] Field, A. P. (2018). Discovering statistics using IBM SPSS statistics (5th ed.). SAGE Publications.

[aur2614-bib-0020] Franklin, J. C. , Ribeiro, J. D. , Fox, K. R. , Bentley, K. H. , Kleiman, E. M. , Huang, X. , Musacchio, K. M. , Jaroszewski, A. C. , Chang, B. P. , & Nock, M. K. (2017). Risk factors for suicidal thoughts and behaviors: A meta‐analysis of 50 years of research. Psychological Bulletin, 143(2), 187–232. 10.1037/bul0000084 27841450

[aur2614-bib-0021] Harris, P. A. , Taylor, R. , Minor, B. L. , Elliott, V. , Fernandez, M. , O'Neal, L. , McLeod, L. , Delacqua, G. , Delacqua, F. , Kirby, J. , & Duda, S. N. (2019). The REDCap consortium: Building an international community of software platform partners. Journal of Biomedical Informatics, 95, 103208. 10.1016/j.jbi.2019.103208 31078660 PMC7254481

[aur2614-bib-0022] Harris, P. A. , Taylor, R. , Thielke, R. , Payne, J. , Gonzalez, N. , & Conde, J. G. (2009). Research electronic data capture (REDCap)—A metadata‐driven methodology and workflow process for providing translational research informatics support. Journal of Biomedical Informatics, 42(2), 377–381. 10.1016/j.jbi.2008.08.010 18929686 PMC2700030

[aur2614-bib-0023] Hedley, D. , & Uljarević, M. (2018). Systematic review of suicide in autism spectrum disorder: Current trends and implications. Current Developmental Disorders Reports, 5, 65–76. 10.1007/s40474-018-0133-6

[aur2614-bib-0024] Hedley, D. , Uljarević, M. , Bury, S. M. , & Dissanayake, C. (2019). Predictors of mental health and well‐being in employed adults with autism spectrum disorder at 12‐month follow‐up. Autism Research, 12, 482–494. 10.1002/aur.2064 30675764

[aur2614-bib-0025] Hedley, D. , Uljarević, M. , Cai, R. Y. , Bury, S. M. , Stokes, M. A. , & Evans, D. W. (2021). Domains of the autism phenotype, cognitive control, and rumination as transdiagnostic predictors of DSM‐5 suicide risk. PLoS One, 16(1), e0245562. 10.1371/journal.pone.0245562 33482664 PMC7822649

[aur2614-bib-0026] Hedley, D. , Uljarević, M. , Foley, K.‐R. , Richdale, A. , & Trollor, J. (2018). Risk and protective factors underlying suicidal ideation in Autism Spectrum disorder. Depression and Anxiety, 35, 648–657. 10.1002/da.22759 29659141

[aur2614-bib-0027] Hedley, D. , Uljarević, M. , Wilmot, M. , Richdale, A. , & Dissanayake, C. (2017). Social support, depression and suicidal ideation in adults with autism spectrum disorder. Journal of Autism and Developmental Disorders, 47(11), 3669–3677. 10.1007/s10803-017-3274-2 28861661

[aur2614-bib-0028] Hedley, D. , Uljarević, M. , Wilmot, M. , Richdale, A. , & Dissanayake, C. (2018). Understanding depression and thoughts of self‐harm in autism: A potential mechanism involving loneliness. Research in Autism Spectrum Disorders, 46, 1–7. 10.1016/j.rasd.2017.11.003

[aur2614-bib-0029] Hirvikoski, T. , Mittendorfer‐Rutz, E. , Boman, M. , Larsson, H. , Lichtenstein, P. , & Bölte, S. (2016). Premature mortality in autism spectrum disorder. The British Journal of Psychiatry, 208(3), 232–238. 10.1192/bjp.bp.114.160192 26541693

[aur2614-bib-0030] Holmes, E. A. , O'Connor, R. C. , Perry, V. H. , Tracey, I. , Wessely, S. , Arseneault, L. , Ballard, C. , Christensen, H. , Cohen Silver, R. , Everall, I. , Ford, T. , John, A. , Kabir, T. , King, K. , Madan, I. , Michie, S. , Przybylski, A. K. , Shafran, R. , Sweeney, A. , … Bullmore, E. (2020). Multidisciplinary research priorities for the COVID‐19 pandemic: A call for action for mental health science. The Lancet Psychiatry, 7(6), 547–560. 10.1016/S2215-0366(20)30168-1 32304649 PMC7159850

[aur2614-bib-0031] Howell, D. C. (2013). Statistical methods for psychology (8th ed.). Thomson Wadsworth.

[aur2614-bib-0032] Hudson, C. C. , Hall, L. , & Harkness, K. L. (2019). Prevalence of depressive disorders in individuals with autism spectrum disorder: A meta‐analysis. Journal of Abnormal Child Psychology, 47(1), 165–175. 10.1007/s10802-018-0402-1 29497980

[aur2614-bib-0033] International Wellbeing Group . (2013). Personal wellbeing index (5th ed.). Deakin University. https://www.acqol.com.au/instruments#measures

[aur2614-bib-0034] Iob, E. , Steptoe, A. , & Fancourt, D. (2020). Abuse, self‐harm and suicidal ideation in the UKduring the COVID‐19 pandemic. British Journal of Psychiatry, 217(4), 543–546. 10.1192/bjp.2020.130 PMC7360935 32654678

[aur2614-bib-0035] Jokiranta‐Olkoniemi, E. , Gyllenberg, D. , Sucksdorff, D. , Suominen, A. , Kronström, K. , Chudal, R. , & Sourander, A. (2021). Risk for premature mortality and intentional self‐harm in Autism Spectrum disorders. Journal of Autism and Developmental Disorders, 51, 3098–3108. 10.1007/s10803-020-04768-x 33140146 PMC8349316

[aur2614-bib-0036] Kavanagh, A. , Dickinson, H. , Carey, G. , Llewellyn, G. , Emerson, E. , Disney, G. , & Hatton, C. (2021). Improving health care for disabled people in COVID‐19 and beyond: Lessons from Australia and England. Disability and Health Journal, 14(2), 101050. 10.1016/j.dhjo.2020.101050 33341397 PMC7969381

[aur2614-bib-0037] Keyes, C. L. (2005). Mental illness and/or mental health? Investigating axioms of the complete state model of health. Journal of Consulting and Clinical Psychology, 73(3), 539–548. 10.1037/0022-006x.73.3.539 15982151

[aur2614-bib-0038] Keyes, C. L. , Shmotkin, D. , & Ryff, C. D. (2002). Optimizing well‐being: The empirical encounter of two traditions. Journal of Personality and Social Psychology, 82(6), 1007–1022. 10.1037/0022-3514.82.6.1007 12051575

[aur2614-bib-0039] Kirby, A. V. , Bakian, A. V. , Zhang, Y. , Bilder, D. A. , Keeshin, B. R. , & Coon, H. (2019). A 20‐year study of suicide death in a statewide autism population. Autism Research, 12(4), 658–666. 10.1002/aur.2076 30663277 PMC6457664

[aur2614-bib-0040] Klonsky, E. D. , Qiu, T. , & Saffer, B. Y. (2017). Recent advances in differentiating suicide attempters from suicide ideators. Current Opinion in Psychiatry, 30(1), 15–20. 10.1097/yco.0000000000000294 27798483

[aur2614-bib-0041] Kõlves, K. , Fitzgerald, C. , Nordentoft, M. , Wood, S. J. , & Erlangsen, A. (2021). Assessment of suicidal behaviors among individuals with autism spectrum disorder in Denmark. JAMA Network Open, 4(1), e2033565. 10.1001/jamanetworkopen.2020.33565 33433599 PMC12578491

[aur2614-bib-0042] Kroenke, K. , & Spitzer, R. L. (2002). The PHQ‐9: A new depression diagnostic and severity measure. Psychiatric Annals, 32, 509–515. 10.3928/0048-5713-20020901-06

[aur2614-bib-0043] Kroenke, K. , Strine, T. W. , Spitzer, R. L. , Williams, J. B. , Berry, J. T. , & Mokdad, A. H. (2009). The PHQ‐8 as a measure of current depression in the general population. Journal of Affective Disorders, 114(1–3), 163–173. 10.1016/j.jad.2008.06.026 18752852

[aur2614-bib-0044] Lai, M.‐C. , Kassee, C. , Besney, R. , Bonato, S. , Hull, L. , Mandy, W. , Szatmari, P. , & Ameis, S. H. (2019). Prevalence of co‐occurring mental health diagnoses in the autism population: A systematic review and meta‐analysis. The Lancet Psychiatry, 6(10), 819–829. 10.1016/S2215-0366(19)30289-5 31447415

[aur2614-bib-0045] Little, R. J. A. (1988). A test of missing completely at random for multivariate data with missing values. Journal of the American Statistical Association, 83(404), 1198–1202. 10.1080/01621459.1988.10478722

[aur2614-bib-0046] Loomes, R. , Hull, L. , & Mandy, W. P. L. (2017). What is the male‐to‐female ratio in autism spectrum disorder? A systematic review and metal‐analysis. Journal of the American Academy of Child and Adolescent Psychiatry, 56(6), 466–474. 10.1016/j.jaac.2017.03.013 28545751

[aur2614-bib-0047] Losada‐Baltar, A. , Jiménez‐Gonzalo, L. , Gallego‐Alberto, L. , Pedroso‐Chaparro, M. D. S. , Fernandes‐Pires, J. , & Márquez‐González, M. (2021). “We are staying at home.” association of self‐perceptions of aging, personal and family resources, and loneliness with psychological distress during the lock‐down period of COVID‐19. Journals of Gerontology, Series B: Psychological Sciiences and Social Sciences, 76(2), e10–e16. 10.1093/geronb/gbaa048 PMC7184373 32282920

[aur2614-bib-0048] Lugo‐Marín, J. , Gisbert‐Gustemps, L. , Setien‐Ramos, I. , Español‐Martín, G. , Ibañez‐Jimenez, P. , Forner‐Puntonet, M. , Arteaga‐Henríquez, G. , Soriano‐Día, A. , Duque‐Yemail, J. D. , & Ramos‐Quiroga, J. A. (2021). COVID‐19 pandemic effects in people with autism spectrum disorder and their caregivers: Evaluation of social distancing and lockdown impact on mental health and general status. Research in Autism Spectrum Disorders, 83, 101757. 10.1016/j.rasd.2021.101757 33649707 PMC7904459

[aur2614-bib-0049] Mars, B. , Heron, J. , Klonsky, E. D. , Moran, P. , O'Connor, R. C. , Tilling, K. , Wilkinson, P. , & Gunnell, D. (2019). Predictors of future suicide attempt among adolescents with suicidal thoughts or non‐suicidal self‐harm: A population‐based birth cohort study. The Lancet Psychiatry, 6(4), 327–337. 10.1016/S2215-0366(19)30030-6 30879972 PMC6494973

[aur2614-bib-0050] Moynihan, R. , Sanders, S. , Michaleff, Z. A. , Scott, A. M. , Clark, J. , To, E. J. , Jones, M. , Kitchener, E. , Fox, M. , Johansson, M. , Lang, E. , Duggan, A. , Scott, I. , & Albarqouni, L. (2021). Impact of COVID‐19 pandemic on utilisation of healthcare services: A systematic review. BMJ Open, 11(3), e045343. 10.1136/bmjopen-2020-045343 PMC7969768 33727273

[aur2614-bib-0051] Newby, J. M. , O'Moore, K. , Tang, S. , Christensen, H. , & Faasse, K. (2020). Acute mental health responses during the COVID‐19 pandemic in Australia. PLoS One, 15(7), e0236562. 10.1371/journal.pone.0236562 32722711 PMC7386645

[aur2614-bib-0052] Nock, M. K. , Green, J. G. , Hwang, I. , McLaughlin, K. A. , Sampson, N. A. , Zaslavsky, A. M. , & Kessler, R. C. (2013). Prevalence, correlates, and treatment of lifetime suicidal behavior among adolescents: Results from the National Comorbidity Survey Replication Adolescent Supplement. JAMA Psychiatry, 70(3), 300–310. 10.1001/2013.jamapsychiatry.55 23303463 PMC3886236

[aur2614-bib-0053] Nock, M. K. , Hwang, I. , Sampson, N. , Kessler, R. C. , Angermeyer, M. , Beautrais, A. , Borges, G. , Bromet, E. , Bruffaerts, R. , de Girolamo, G. , de Graaf, R. , Florescu, S. , Gureje, O. , Haro, J. M. , Hu, C. , Huang, Y. , Karam, E. G. , Kawakami, N. , Kovess, V. , … Williams, D. R. (2009). Cross‐national analysis of the associations among mental disorders and suicidal behavior: Findings from the WHO world mental health surveys. PLoS Medicine, 6(8), e1000123. 10.1371/journal.pmed.1000123 19668361 PMC2717212

[aur2614-bib-0054] O'Connor, R. C. , Wetherall, K. , Cleare, S. , McClelland, H. , Melson, A. J. , Niedzwiedz, C. L. , O'Carroll, R. E. , O'Connor, D. B. , Platt, S. , Scowcroft, E. , Watson, B. , Zortea, T. , Ferguson, E. , & Robb, K. A. (2021). Mental health and well‐being during the COVID‐19 pandemic: Longitudinal analyses of adults in the UK COVID‐19 Mental Health & Wellbeing study. The British Journal of Psychiatry, 218(6), 326–333. 10.1192/bjp.2020.212 33081860 PMC7684009

[aur2614-bib-0055] Oomen, D. , Nijhof, A. D. , & Wiersema, J. R. (2021). The psychological impact of the COVID‐19 pandemic on adults with autism: A survey study across three countries. Molecular Autism, 12(1), 21. 10.1186/s13229-021-00424-y 33658046 PMC7927758

[aur2614-bib-0056] Osman, A. , Bagge, C. L. , Gutierrez, P. M. , Konick, L. C. , Kopper, B. A. , & Barrios, F. X. (2001). The suicidal behaviors questionnaire‐revised (SBQ‐R): Validation with clinical and nonclinical samples. Assessment, 8(4), 443–454. 10.1177/107319110100800409 11785588

[aur2614-bib-0057] Pierce, M. , Hope, H. , Ford, T. , Hatch, S. , Hotopf, M. , John, A. , Kontopantelis, E. , Webb, R. , Wessely, S. , McManus, S. , & Abel, K. M. (2020). Mental health before and during the COVID‐19 pandemic: A longitudinal probability sample survey of the UKpopulation. The Lancet Psychiatry, 7(10), 883–892. 10.1016/S2215-0366(20)30308-4 32707037 PMC7373389

[aur2614-bib-0058] Pirkis, J. , John, A. , Shin, S. , DelPozo‐Banos, M. , Arya, V. , Analuisa‐Aguilar, P. , Appleby, L. , Arensman, E. , Bantjes, J. , Baran, A. , Bertolote, J. M. , Borges, G. , Brečić, P. , Caine, E. , Castelpietra, G. , Chang, S.‐S. , Colchester, D. , Crompton, D. , Curkovic, M. , … Spittal, M. J. (2021). Suicide trends in the early months of the COVID‐19 pandemic: An interrupted time‐series analysis of preliminary data from 21 countries. The Lancet Psychiatry, 8(7), 579–588. 10.1016/S2215-0366(21)00091-2 33862016 PMC9188435

[aur2614-bib-0059] Ramiz, L. , Contrand, B. , Rojas Castro, M. Y. , Dupuy, M. , Lu, L. , Sztal‐Kutas, C. , & Lagarde, E. (2021). A longitudinal study of mental health before and during COVID‐19 lockdown in the French population. Globalization and Health, 17(1), 29. 10.1186/s12992-021-00682-8 33752717 PMC7982911

[aur2614-bib-0060] Salari, N. , Hosseinian‐Far, A. , Jalali, R. , Vaisi‐Raygani, A. , Rasoulpoor, S. , Mohammadi, M. , Rasoulpoor, S. , & Khaledi‐Paveh, B. (2020). Prevalence of stress, anxiety, depression among the general population during the COVID‐19 pandemic: A systematic review and meta‐analysis. Globalization and Health, 16(1), 57. 10.1186/s12992-020-00589-w 32631403 PMC7338126

[aur2614-bib-0061] Salkind, N. J. (2007). Encyclopedia of measurement and statistics. Sage Publications, Inc. 10.4135/9781412952644

[aur2614-bib-0062] Shin, C. , Lee, S. H. , Han, K. M. , Yoon, H. K. , & Han, C. (2019). Comparison of the usefulness of the PHQ‐8 and PHQ‐9 for screening for major depressive disorder: Analysis of psychiatric outpatient data. Psychiatry Investigation, 16(4), 300–305. 10.30773/pi.2019.02.01 31042692 PMC6504773

[aur2614-bib-0063] Sokoloff, W. C. , Krief, W. I. , Giusto, K. A. , Mohaimin, T. , Murphy‐Hockett, C. , Rocker, J. , & Williamson, K. A. (2021). Pediatric emergency department utilization during the COVID‐19 pandemic in new York City. American Journal of Emergency Medicine, 45, 100–104. 10.1016/j.ajem.2021.02.029 33677263 PMC7896495

[aur2614-bib-0064] Stanley, I. H. , Day, T. N. , Gallyer, A. J. , Shelef, L. , Kalla, C. , Gutierrez, P. M. , & Joiner, T. E. (2021). Autism‐related traits and suicide risk among active duty U.S. military service members. Psychological Services, 18(3), 377–388. 10.1037/ser0000418 32105121

[aur2614-bib-0065] Stoddard, J. , & Kaufman, J. (2020). Coronavirus impact scale . https://www.phenxtoolkit.org/toolkit_content/PDF/CIS_Stoddard.pdf

[aur2614-bib-0066] Stoddard, J. , Reynolds, E. K. , Paris, R. , Haller, S. , Johnson, S. , Zik, J. , Elliotte, E. , Maru, M. , Jaffe, A. , Mallidi, A. , Smith, A. , Hernandez, R. G. , Volk, H. E. , Brotman, M. A. , & Kaufman, J. (2021, The coronavirus impact scale: Construction, validation, and comparisons in diverse clinical samples. 10.31234/osf.io/kz4pg PMC10010775 37359142

[aur2614-bib-0067] Tabachnick, B. G. , & Fidell, L. S. (2007). Using multivariate statistics (5th ed.). Allyn & Bacon.

[aur2614-bib-0068] Tan, E. J. , Meyer, D. , Neill, E. , Phillipou, A. , Toh, W. L. , Van Rheenen, T. E. , & Rossell, S. L. (2020). Considerations for assessing the impact of the COVID‐19 pandemic on mental health in Australia. Australian & New Zealand Journal of Psychiatry, 54(11), 1067–1071. 10.1177/0004867420947815 32746614 PMC7404085

[aur2614-bib-0069] Tanaka, T. , & Okamoto, S. (2021). Increase in suicide following an initial decline during the COVID‐19 pandemic in Japan. Nature Human Behaviour, 5(2), 229–238. 10.1038/s41562-020-01042-z 33452498

[aur2614-bib-0070] Teismann, T. , Brailovskaia, J. , Siegmann, P. , Nyhuis, P. , Wolter, M. , & Willutzki, U. (2018). Dual factor model of mental health: Co‐occurrence of positive mental health and suicide ideation in inpatients and outpatients. Psychiatry Research, 260, 343–345. 10.1016/j.psychres.2017.11.085 29232575

[aur2614-bib-0071] Teismann, T. , Forkmann, T. , Brailovskaia, J. , Siegmann, P. , Glaesmer, H. , & Margraf, J. (2018). Positive mental health moderates the association between depression and suicide ideation: A longitudinal study. International Journal of Clinical and Health Psychology, 18(1), 1–7. 10.1016/j.ijchp.2017.08.001 30487904 PMC6220923

[aur2614-bib-0072] Thorpe, K. G. (2018). *The subjective wellbeing of Australians with an Autism Spectrum condition* [Master's dissertation, Deakin University, Melbourne, Australia. http://hdl.handle.net/10536/DRO/DU:30112386

[aur2614-bib-0073] Uljarević, M. , Hedley, D. , Cai, R.‐Y. , Hardan, A. Y. , & South, M. (2020). Anxiety and depression from adolescence to old age in Autism Spectrum disorder. In F. R. Volkmar (Ed.), Encyclopedia of autism spectrum disorders. Springer. 10.1007/978-1-4614-6435-8_102432-1 31190198

[aur2614-bib-0074] Viner, R. M. , Russell, S. J. , Croker, H. , Packer, J. , Ward, J. , Stansfield, C. , Mytton, O. , Bonell, C. , & Booy, R. (2020). School closure and management practices during coronavirus outbreaks including COVID‐19: A rapid systematic review. The Lancet Child & Adolescent Health, 4(5), 397–404. 10.1016/S2352-4642(20)30095-X 32272089 PMC7270629

[aur2614-bib-0075] World Health Organization . (2021). *WHO Health Emergency Dashboard WHO (COVID‐19) Homepage: Global: Australia*. World Health Organization. https://covid19.who.int/region/wpro/country/au

