# Supplementary material for: The association between COVID‐19, personal wellbeing, depression, and suicide risk factors in Australian autistic adults
Source: Autism Res. 2021 Sep 21;14(12):2663–76. doi: 10.1002/aur.2614 (PMC8646719; doi:10.1002/aur.2614)
Supplement: Supplementary file 1 — Table S1 Bootstrapped Bonferroni corrected multiple comparisons for COVID‐19 impact between Australian states and territories [file AUR-14-2663-s002.docx]

**Supplementary Table S1. Bootstrapped Bonferroni corrected multiple comparisons for COVID-19 impact between Australian states and territories**

| **State/**  **Territory** | **Comparison** | **Mean Difference** | **Bias** | **Std. Error** | **BCa 95% Confidence Interval** | |  |
| --- | --- | --- | --- | --- | --- | --- | --- |
|  |  |  |  |  | **Lower** | **Upper** | **Cohen’s *d* [95%CI)** |
| NSW | ACT | -.157 | -.014^b^ | 1.444^b^ | -3.022^b,c^ | 2.771^b^ | 0.038 [-0.738,0.813] |
|  | VIC | -1.983 | .027^b^ | 1.352^b^ | -4.586^b,c^ | .722^b^ | 0.399 [-0.149,0.947] |
|  | QLD | 1.364 | -.012^b^ | 1.423^b^ | -1.370^b,c^ | 4.093^b^ | 0.286 [-0.308,0.880] |
|  | SA | 2.455 | .003^d^ | 1.240^d^ | .133^c,d^ | 4.907^d^ | 0.567 [-0.509,1.644] |
|  | WA | 1.732 | .034^b^ | 1.799^b^ | -2.035^b,c^ | 5.030^b^ | 0.379 [-0.402,1.160] |
|  | TAS | 4.205 | .012^e^ | 1.626^e^ | .702^c,e^ | 7.097^e^ | 0.955 [-0.141,2.052] |
| ACT | NSW | .157 | .014^b^ | 1.444^b^ | -2.771^b,c^ | 3.022^b^ | – |
|  | VIC | -1.826 | .041^b^ | 1.447^b^ | -4.690^b,c^ | .986^b^ | 0.373 [-0.371,1.117] |
|  | QLD | 1.520 | .002^b^ | 1.496^b^ | -1.510^b,c^ | 4.423^b^ | 0.336 [-0.444,1.116] |
|  | SA | 2.611 | .013^d^ | 1.335^d^ | .034^c,d^ | 5.330^d^ | 0.903 [-0.325,2.131] |
|  | WA | 1.889 | .048^b^ | 1.867^b^ | -1.966^b,c^ | 5.397^b^ | 0.480 [-0.458,1.417] |
|  | TAS | 4.361 | .026^e^ | 1.706^e^ | .714^c,e^ | 7.500^e^ | 1.394 [0.100,2.688] |
| VIC | NSW | 1.983 | -.027^b^ | 1.352^b^ | -.722^b,c^ | 4.586^b^ | – |
|  | ACT | 1.826 | -.041^b^ | 1.447^b^ | -.986^b,c^ | 4.690^b^ | – |
|  | QLD | 3.347 | -.039^b^ | 1.405^b^ | .536^b,c^ | 6.075^b^ | 0.654 [-0.098.1.211] |
|  | SA | 4.438 | -.026^d^ | 1.220^d^ | 2.113^c,d^ | 6.952^d^ | 0.881 [-0.178,1.941] |
|  | WA | 3.715 | .007^b^ | 1.778^b^ | .090^b,c^ | 7.111^b^ | 0.728 [-0.029,1.484] |
|  | TAS | 6.188 | -.013^e^ | 1.630^e^ | 2.750^c,e^ | 9.053^e^ | 1.218 [0.144,2.295] |
| QLD | NSW | -1.364 | .012^b^ | 1.423^b^ | -4.093^b,c^ | 1.370^b^ | – |
|  | ACT | -1.520 | -.002^b^ | 1.496^b^ | -4.423^b,c^ | 1.510^b^ | – |
|  | VIC | -3.347 | .039^b^ | 1.405^b^ | -6.075^b,c^ | -.536^b^ | – |
|  | SA | 1.091 | .014^d^ | 1.321^d^ | -1.444^c,d^ | 3.755^d^ | 0.235 [-0.833,1.302] |
|  | WA | .369 | .046^b^ | 1.891^b^ | -3.614^b,c^ | 3.872^b^ | 0.077 [-0.699,0.853] |
|  | TAS | 2.841 | .024^e^ | 1.712^e^ | -.800^c,e^ | 5.960^e^ | 0.602 [-0.476,1.680] |
| SA | NSW | -2.455 | -.003^d^ | 1.240^d^ | -4.907^c,d^ | -.133^d^ | – |
|  | ACT | -2.611 | -.013^d^ | 1.335^d^ | -5.330^c,d^ | -.034^d^ | – |
|  | VIC | -4.437 | .026^d^ | 1.220^d^ | -6.952^c,d^ | -2.113^d^ | – |
|  | QLD | -1.091 | -.014^d^ | 1.321^d^ | -3.755^c,d^ | 1.444^d^ | – |
|  | WA | -.722 | .034^d^ | 1.717^d^ | -4.375^c,d^ | 2.342^d^ | 0.181 [-0.999,1.361] |
|  | TAS | 1.750 | .013^f^ | 1.546^f^ | -1.716^c,f^ | 4.300^f^ | 0.738 [-0.694,2.170] |
| WA | NSW | -1.732 | -.034^b^ | 1.799^b^ | -5.030^b,c^ | 2.035^b^ | – |
|  | ACT | -1.889 | -.048^b^ | 1.867^b^ | -5.397^b,c^ | 1.966^b^ | – |
|  | VIC | -3.715 | -.007^b^ | 1.778^b^ | -7.111^b,c^ | -.090^b^ | – |
|  | QLD | -.369 | -.046^b^ | 1.891^b^ | -3.872^b,c^ | 3.614^b^ | – |
|  | SA | .722 | -.034^d^ | 1.717^d^ | -2.342^c,d^ | 4.375^d^ | – |
|  | TAS | 2.472 | -.027^e^ | 2.051^e^ | -1.625^c,e^ | 6.500^e^ | 0.594 [-0.606,1.794] |
| TAS | NSW | -4.205 | -.012^e^ | 1.626^e^ | -7.097^c,e^ | -.702^e^ | – |
|  | ACT | -4.361 | -.026^e^ | 1.706^e^ | -7.500^c,e^ | -.714^e^ | – |
|  | VIC | -6.187 | .013^e^ | 1.630^e^ | -9.053^c,e^ | -2.750^e^ | – |
|  | QLD | -2.841 | -.024^e^ | 1.712^e^ | -5.960^c,e^ | .800^e^ | – |
|  | SA | -1.750 | -.013^f^ | 1.546^f^ | -4.300^c,f^ | 1.716^f^ | – |
|  | WA | -2.472 | .027^e^ | 2.051^e^ | -6.500^c,e^ | 1.625^e^ | – |
