# Supplementary material for: The association between COVID‐19, personal wellbeing, depression, and suicide risk factors in Australian autistic adults
Source: Autism Res. 2021 Sep 21;14(12):2663–76. doi: 10.1002/aur.2614 (PMC8646719; doi:10.1002/aur.2614)
Supplement: Supplementary file 2 — Table S2 Linear regression model of predictors of SBQ‐R suicide risk scores with gender (binary variable) entered into the model (n = 94) [file AUR-14-2663-s001.docx]

**Supplementary Table S2. Linear regression model of predictors of SBQ-R suicide risk scores with gender (binary variable) entered into the model (*n =* 94)**

|  |  | ***b*** | ***SEB***^a^ | ***β*** | ***p*-value** | ***BCa 95% CI*** |
| --- | --- | --- | --- | --- | --- | --- |
| Step 1 | Constant | 9.257 | .576 | – | <.001 | **8.11, 10.38** |
|  | Gender | .997 | .774 | .127 | .196 | -0.543, 2.609 |
| Step 2 | Constant | 13.574 | 1.807 | – | <.001 | **9.98, 17.21** |
|  | Gender | .962 | .655 | .123 | .147 | -0.325, 2.34 |
|  | COVID-19 | -0.066 | .074 | -0.082 | .377 | -0.219, 0.062 |
|  | Personal Wellbeing | -0.091 | .021 | -0.502 | <.001 | **-0.131, -0.051** |
|  | Depression | 0.091 | .077 | 0.144 | .239 | -0.061, 0.256 |

Note. Step 1, *R^2^* = .016, *F*(1, 92) = 1.52, *p =* .221; Step 2, *R^2^Δ* = .359, *F*(3, 89) = 13.38, *p <* .001. BCa 95% confidence intervals that do not cross zero are bolded. *P*-values and 95% bias corrected and accelerated confidence intervals and standard errors based on 5000 bootstrap samples.

^a^SEB: the standard error for the unstandardized beta.
